# Primary Aortoduodenal Fistula: First you Should Suspect it

**DOI:** 10.5935/1678-9741.20160049

**Published:** 2016

**Authors:** Mircea Beuran, Ionut Negoi, Ruxandra Irina Negoi, Sorin Hostiuc, Sorin Paun

**Affiliations:** 1 Carol Davila University of Medicine and Pharmacy Bucharest, General Surgery Department, Emergency Hospital of Bucharest, Romania.; 2 Carol Davila University of Medicine and Pharmacy Bucharest, Romania.; 3 Carol Davila University of Medicine and Pharmacy Bucharest, Mina Minovici National Institute of Legal Medicine, Romania.

**Keywords:** Aorta, Abdominal, Duodenum, Digestive System Fistula

## Abstract

A 59 year-old patient was admitted with upper gastrointestinal bleeding. The clinical exam showed mild hypotension and blood samples revealed acute anemia (hemoglobin = 7.5 g/dl). Emergency computed tomography showed an infrarenal abdominal aortic aneurysm and extravasation of the arterial contrast material toward the digestive tract. The patient was transported to the operating room for emergency laparotomy, which showed an aortoduodenal fistula. After proximal and distal aortic vascular control, the two anatomical structures were dissected with duodenorrhaphy, patch repair of the aortic tear and omentum interposition. The postoperative recovery was uneventful, with discharge after 12 days.

**Table:** 

**Abbreviations, acronyms & symbols**	
CT	= Computed tomography

## INTRODUCTION

Aortoenteric fistulas represent a rare but life-threating condition, with an annual incidence of 0.007 per million. In the scientific literature, there are about 350 cases of primary aortoenteric fistulas, fewer than 200 primary aortoduodenal fistulas, and a total of 791 primary and secondary aortoduodenal fistulas^[1]^. Autopsy reports suggest an incidence of 0.02%-0.07% of primary fistulas and 1% in patients with abdominal aorta reconstructions^[2]^. Due to its close proximity with the abdominal aorta, the third and fourth duodenum represent the most common involved digestive segments in aortoenteric fistulas, followed by jejunum and ileum. The aortoenteric fistulas are classified into primary, in which the digestive tract is compressed by an aortic aneurysm, and secondary fistulas, produced by erosion of an aortic prosthesis into the digestive tract. Mortality is very high, being 100% in untreated patients and 30-40% in surgically approached fistulas^[3]^. The clinical picture of aortoenteric fistulas is characterized by a 'herald gastrointestinal bleeding', with hematemesis and melena, followed by severe bleeding and exsanguination. In clinical practice, there must be a high level of suspicion combined with emergency computed tomography (CT) in order to allow a timely surgical approach with or without a preoperative endovascular access. Despite current imagistic advances, up to two thirds of the primary aortoenteric fistulas are diagnosed on exploratory laparotomy.

The objective of this case report is to illustrate a rare cause of upper gastrointestinal bleeding, which can be successfully managed. Written informed consent for publishing the medical data and ethical approval of the hospital's review board were obtained.

## CASE REPORT

**Fig. 1:**
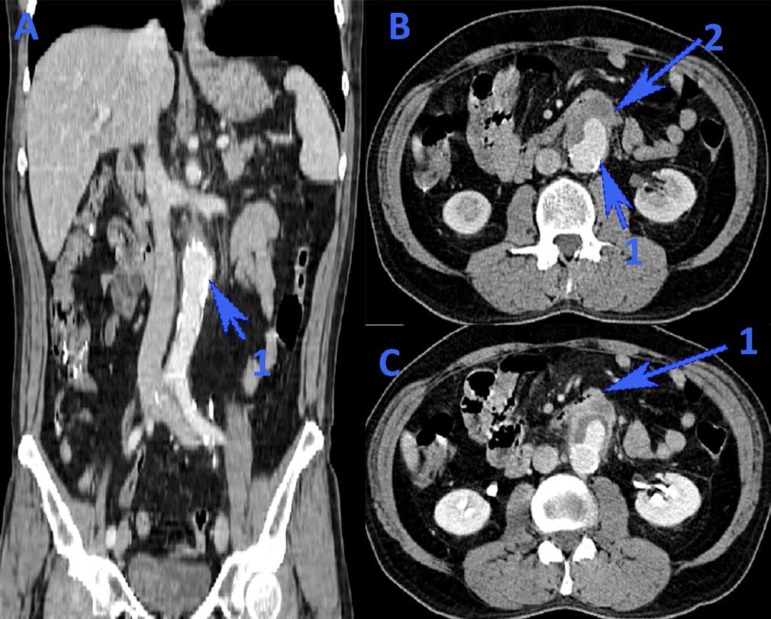
Computed tomography images. A – Coronal view showing the infrarenal abdominal aortic aneurysm (1); B – Anterior to the aortic aneurysm (1), a pseudoaneurysm (2) can be observed in close contact with the third and fourth duodenum (1 in image C).

A 59 year-old patient was referred to our hospital for upper gastrointestinal bleeding, revealed by hematemesis and melena. On clinical exam, the patient was mildly hypotensive (110/60 mmHg), with significant paleness. Blood samples revealed acute anemia (hemoglobin = 7.5 g/dl), and emergency upper gastrointestinal endoscopy revealed a hiatal hernia and antral gastritis, with no visible source of bleeding. Lower gastrointestinal endoscopy revealed no significant pathologies up to the final 10 cm of the ileum. An abdominal ultrasonography showed communication between the abdominal aorta, below the origin of the renal arteries, and an anterior pseudoaneurysm, with a diameter of 4.7/3.5 cm and orifice of 5.4 mm. Emergency CT showed an infrarenal abdominal aortic aneurysm, 5.5 cm distal to the renal arteries emergence, with thick walls and edema (Figure 1). Anterior to the aneurysmal aortic area, a 45/35 mm pseudoaneurysm in close contact with a small bowel loop was observed, together with extravasation of the arterial contrast material into the digestive tract. There was no free retroperitoneal fluid.

**Fig. 2:**
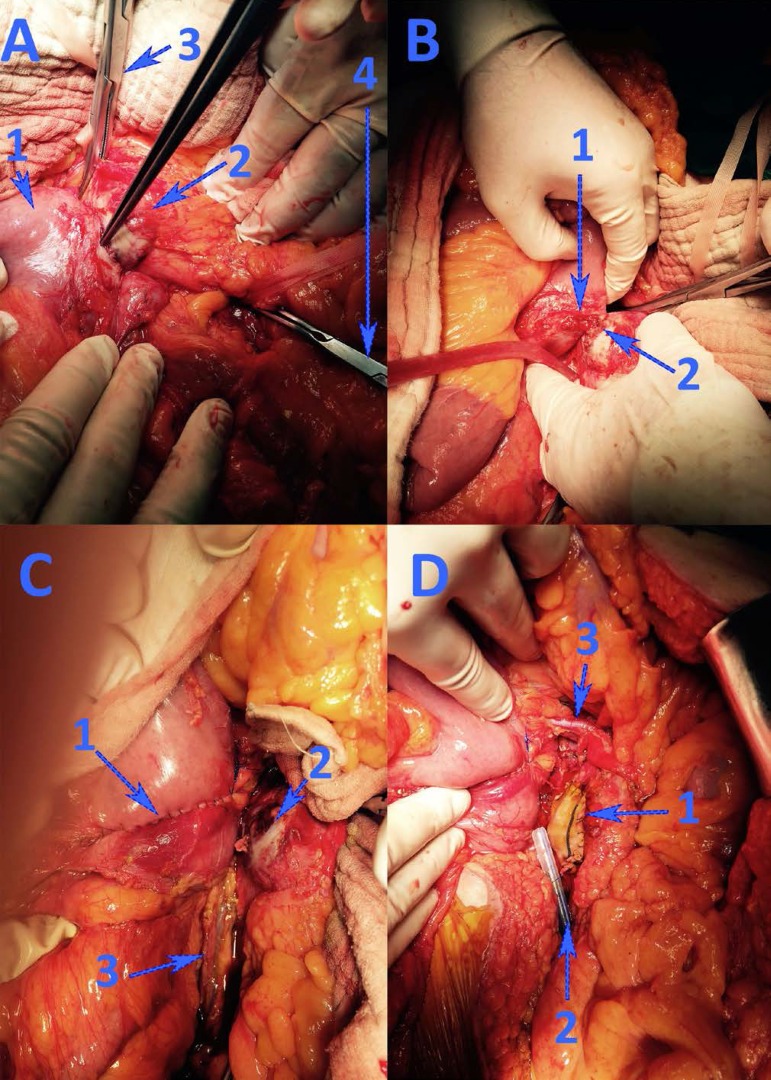
Intraoperative aspects. Image A: 1 – fourth duodenum; 2 – aortic aneurysm; 3 – proximal aortic clamping; 4 – distal aortic clamping. Image B: 1 – duodenal and 2 – aortic holes, after fistula dissection. Image C: 1 – duodenorrhaphy; 2 – aortic aneurysm; 3 – inferior mesenteric vein. Image D: 1 – Dacron aortic patch repair; 2 – local drainage tube; 3 – left renal vein.

The patient was transported to the operating room for emergency laparotomy. After extensive Kocher and Catell-Brasch maneuvers, the aortic aneurysm was exposed in close contact with the fourth duodenum (Figure 2). The upper pole of the aortic aneurysm was located 5 below the origin of the renal arteries. After proximal and distal aortic vascular control, the fourth duodenum was dissected from the aneurysmal area, and a 5 mm communication was identified between the two anatomical structures. The duodenal defect was closed with a continuous, double layer suture. The aortic aneurysmal tear was closed using a Dacron patch, which was covered with biological glue and an omental pedicle. The postoperative recovery was uneventful, with a restart of the oral diet in the fifth postoperative day and discharge after 12 days.

## DISCUSSION

We presented the case of a successfully managed primary aortoduodenal fistula, based on high resolution imaging and a multidisciplinary surgical team.

The primary aortoduodenal fistulas are clinically revealed by gastrointestinal bleeding in approximately 80% of the cases, abdominal pain in 32% of the patients, and a pulsating abdominal mass in 25% of the cases. The upper gastrointestinal bleeding is usually self-limited, with a secondary massive hemorrhage within the next six hours in one-third of the cases^[4]^.

One of the most suggestive CT signs for aortoenteric fistulas is ectopic gas outside the intestinal lumen. It should be noted that ectopic gas may be found up to one month after normal aortic graft repairs and perigraft fluid can be identified up to three months later. Hagspiel et al.^[5]^ published the results of CT angiography in nine patients, one with primary and eight with secondary aortoenteric fistulas. Primary CTs were present in only 3 (33%) cases, showing active extravasation of the aortic contrast into the bowel lumen in one (11%) case and migration of the aortic graft within the bowel lumen in two (22%) cases. Secondary signs, which suggest aortoenteric fistulas but may also be present in patients with graft infection, were: absence of periaortic or perigraft fat planes (100%), thickening of the bowel wall located in contact with the graft (89%), free fluid surrounding the graft (78%), and ectopic gas (56%)^[5]^.

Therapeutic approaches in patients with primary aortoenteric fistula may be either open surgery or endovascular repair. Menezes et al.^[6]^ showed that there are no differences in overall mortality when comparing endovascular with open aortic aneurysm repair (7.69% *versus* 11.89%, *P*=0.263). However, patients with a ruptured or inflammatory aortic aneurysm were excluded from this cohort^[6]^. In our case, we decided for an emergency open approach instead of endovascular repair due to significant contrast extravasation on CT and patient's decreased physiological reserve in case of a failed endovascular approach. Burks et al.^[7]^ published their five-year experience in endovascular repair of bleeding aortoenteric fistulas. Out of seven patients managed with coil embolization (one) or endovascular stent graft (six), three (43%) were alive at a mean of 36 months (range, 23-67 months) after the procedure. There was one perioperative death due to fungal sepsis, persistent sepsis that required laparotomy and bowel resection in one patient, and one patient with recurrent sepsis managed by antibiotic therapy^[7]^. A Greek multicenter study compared open (17 patients) and endovascular (8 patients) repair of secondary aortoenteric fistulas (mean of four years after initial aortic surgery)^[8]^. Although endovascular repair was associated with a lower early morbidity and mortality, the long-term survival rates were similar after two years, due to sepsis and/or recurrence of the fistula in the endovascular group^[8]^.

The objectives of the open surgery are proximal and distal aortic control, management of the intestinal defect and aortic repair. Reviewing the operative results of 81 primary aortoenteric fistulas, managed between 1818 and 1998, Lee et al.^[9]^ found a survival rate of 77.35% for *in situ* grafting, 11.76% for extraanatomic bypass, 100% for aneurysmorrhaphy, and 62.5% for rifampicin-soaked patch usage. We chose the patch aortoplasty due to the simplicity of the method, with favorable short-term results and low postoperative morbidity. The two-step approach was considered safer, with high risk of infection for a definitive emergency aortic aneurysmal repair concomitant with the duodenal closure. After postoperative recovery, the patient was scheduled for elective aneurysmal repair in the cardiovascular service.

Lastly, Rodrigues dos Santos et al.^[1]^ analyzed the results of surgical repair in 791 cases of aortoduodenal fistula. The results of the multivariate analysis revealed that omentum interposition is the strongest independent predictor of survival. The most common cause of death is fistula recurrence (41.8%), which is significantly higher in patients with simple duodenorrhaphy^[1]^.

## CONCLUSION

Only an appropriate diagnostic workup based on a high level of suspicion can offer a chance of survival in patients with aortoduodenal fistulas. Emergency referral to a tertiary center with appropriate material and human resources may decrease the morbidity and mortality of these patients.

**Table:** 

**Authors' roles & responsibilities**	
MB	Analysis and/or data interpretation; final manuscript approval
IN	Analysis and/or data interpretation; manuscript writing or critical review of its content; final manuscript approval
RIN	Manuscript writing or critical review of its content; final manuscript approval
SH	Conception and study design; final manuscript approval
SP	Conception and study design; final manuscript approval
